# Shenmayizhi Formula Combined with Ginkgo Extract Tablets for the Treatment of Vascular Dementia: A Randomized, Double-Blind, Controlled Trial

**DOI:** 10.1155/2020/8312347

**Published:** 2020-07-25

**Authors:** Huiqin Zhang, Yu Cao, Hui Pei, Huichan Wang, Lina Ma, Zhiyong Wang, Xuemei Diao, Yang Yang, Nanyang Liu, Yun Wei, Hao Li

**Affiliations:** ^1^Department of Geriatrics, Xiyuan Hospital, China Academy of Chinese Medical Sciences, Beijing 100091, China; ^2^Department of TCM, The Second People's Hospital of Yunnan Province, Yunnan 650021, China

## Abstract

Shenmayizhi formula (SMYZF) has been shown to have an effect on vascular dementia (VaD) in previous studies. The aim of this study was to evaluate whether a combination of SMYZF with Ginkgo extract tablets improves mild-to-moderate VaD. In this 12-week, randomized, double-blind, controlled study, we randomly assigned 196 patients with VaD (aged 50–85 years) to either the SMYZF group (*n* = 98) or the Ginkgo group (*n* = 98). All patients received Ginkgo extract tablets as a basic treatment, while the SMYZF group also received SMYZF treatment. We evaluated the participants at baseline and after 12 weeks of the intervention for the following: the Mini-Mental State Examination (MMSE), National Institutes of Health Stroke Scale (NIHSS), activities of daily living (ADL), Chinese Medicine Symptom Scale (CM-SS) scores, serum endothelin-1 (ET-1), nitric oxide (NO), vascular endothelial growth factor (VEGF), von Willebrand factor (vWF), neuron-specific enolase (NSE), brain-derived neurotrophic factor (BDNF), and homocysteine (Hcy) serum levels. Both interventions significantly increased MMSE scores and decreased NIHSS, ADL, and CM-SS scores. The SMYZF group showed greater improvement in MMSE, NIHSS, and CM-SS scores. Both groups showed a significant decrease in serum ET-1 and an increase in serum VEGF. Furthermore, serum NO increased, and vWF decreased significantly in the SMYZF group. Changes in serum ET-1 and NO were greater in the SMYZF group. Both groups showed a significant increase in serum BDNF and a decrease in serum NSE and Hcy. Improvement in serum NSE and BDNF was greater in the SMYZF group. SMYZF combined with Ginkgo extract tablets improved vascular endothelial and cognitive functions, as well as the syndromes diagnosed based on the traditional Chinese medicine in patients with VaD.

## 1. Introduction

The prevalence of dementia is expected to increase because of a rapidly aging population. Dementia imposes a considerable burden on patients, healthcare providers, and the society [1–3], unless its onset can be delayed [4, 5]. After Alzheimer's disease, vascular dementia (VaD) is the most common type of dementia in the world and is mainly caused by hemorrhagic or ischemic cerebrovascular disease. It is characterized by cognitive impairment accompanied by neurodegeneration. It accounts for about 15–20% of dementia cases in North America and Europe [6–9] and about 30% in Asia and developing countries [10–12]. Currently, clinical VaD treatment mainly focuses on improving neurological function and blood circulation in the brain [13, 14]. The effects of vascular pathology and neurodegeneration on cognitive decline appear to be synergistic [15]; therefore, improving vascular endothelial function and reducing neurodegeneration are necessary for prevention of early VaD. Currently, a successful drug treatment for VaD does not exist [16, 17].

Recently, herbal preparations (e.g., Ginkgo extracts and Ginseng products) have been proposed as treatments for memory loss in the elderly [18]. Furthermore, natural products and herbal medicines are widely used for the management and treatment of central nervous system disorders [14]. Herbal medicines are mainly characterized as safe, with few side effects and suitable efficacy. Thus, traditional Chinese medicine (TCM) may have advantages in the treatment of VaD.

In TCM, VaD is thought to be closely associated with the brain and kidney, and its main treatments include tonifying the kidney and essence, activating blood circulation, eliminating phlegm, opening orifices, and removing blood stasis. Currently, however, there is no specific TCM prescription for VaD. Previous studies have indicated that Shenmayizhi formula (SMYZF) could protect nerve cells and preserve vascular endothelial function [19, 20]; however, these studies involved limited preclinical observation, and there is a need to verify its efficacy further.

Additionally, many previous studies have reported that Ginkgo extracts could inhibit apoptosis of hippocampal neurons and protect nerves [21, 22]. Ginkgo extract tablets have also been reported to increase cerebral blood flow, improve hemodynamics, and protect brain function in patients with VaD [23–25].

Therefore, in this study, we used Ginkgo extract tablets as the basic treatment drug and used a large sample size to evaluate the clinical efficacy of SMYZF for improving vascular endothelial function and neurological deficits in patients with mild-to-moderate VaD. Specifically, we assessed pre- and posttreatment neuropsychological scale scores and related serum indexes to evaluate the clinical efficacy of SMYZF.

## 2. Materials and Methods

### 2.1. Trial Design

This was a 12-week, randomized, double-blind, controlled trial to evaluate the clinical efficacy of SMYZF combined with Ginkgo extract tablets, which was undertaken in the Community Healthcare Centers of the Jingzhuang town and Shenjiaying, Yanqing District, Beijing, from June 2018 to January 2019. The protocol was approved by the Ethics Committee of Xiyuan Hospital, Chinese Academy of Chinese Medical Sciences (2017XLA033-3). Furthermore, an external data and safety monitoring committee was set up for this study, and the trial was registered with the China Clinical Trials Registry (ChiCTR1800017359).

### 2.2. Participants

The inclusion criteria were as follows: (1) age between 50 and 85 years; (2) fulfillment of diagnostic criteria for VaD according to the Diagnostic and Statistical Manual of Mental Disorders [26] and the National Institute of Neurological Disorders and Stroke [27]; (3) Clinical Dementia Rating (CDR) score of 1 or 2 (defined as mild-to-moderate cognitive impairment) [28]; (4) Mini-Mental State Examination (MMSE) score of 10–26; (5) Hachinski Ischemic Scale (HIS) score ≥ 7; (6) National Institutes of Health Stroke Scale (NIHSS) score of 5–15; and (7) provision of written informed consent. The exclusion criteria were as follows: (1) HIS score ≤ 6; (2) dementia complicated with depression (reference: Hamilton Depression Scale (HAMD)); (3) CDR score = 3; (4) hemorrhagic or transient cerebral ischemia, severe neurological impairment such as dominant hand hemiplegia, audio-visual impairment, or aphasia, and severe heart, liver, or kidney function impairment; and (5) recent use (within 3 months) of drugs that might affect cognitive function or allergy to the drugs used in this study.

### 2.3. Randomization and Blinding

The Good Clinical Practice Center of Xiyuan Hospital was responsible for computer generation of the allocation sequence for block randomization of participants. Beijing Kangrentang Pharmaceutical Company prepared and provided the SMYZF granule packages and placebo according to the randomization assignment. All patients were randomly assigned either to the Ginkgo group (*n* = 98) or the SMYZF group (*n* = 98) in a 1 : 1 ratio; all patients in both groups received Ginkgo extract tablets, as well as either placebo or SMYZF granules, respectively. All the collaborating investigators and participants were blinded to the treatment allocation of the patients.

### 2.4. Intervention

Patients in both groups took Ginkgo extract tablets (1 tablet, orally, three times daily; obtained from Yangzijiang Pharmaceutical Co., Ltd., Nanjing, China; batch no. 10010321) as a basic treatment. The patients in the SMYZF group took SMYZF granules (4.8 g, orally, twice daily; obtained from Beijing Kangrentang Pharmaceutical Co. Ltd.; Beijing, China), which comprised Radix et Rhizoma Ginseng, Rhizoma Gastrodiae, Ramuli Euonymi, and Rhizoma Chuanxiong. The patients in the Ginkgo group took 4.8 g SMYZF-placebo granules (4.8 g, orally, twice daily; obtained from Beijing Kangrentang Pharmaceutical Co., Ltd.; Beijing, China), which comprised 5% SMYZF granules + 95% dextrin. The taste of the placebo and SMYZF granules was similar. The course of treatment was 12 weeks.

### 2.5. Serum Analyses

We obtained blood samples at baseline and at the endpoint. After centrifugation, the upper serum layer was transferred to a disposable EP tube and frozen in a refrigerator at −80°C until required. The serum samples were then thawed at room temperature prior to analysis.

The serum indexes related to vascular endothelial function were determined, which included serum endothelin-1 (ET-1), nitric oxide (NO), vascular endothelial growth factor (VEGF), and von Willebrand factor (vWF). Serum indexes related to neurological function were also determined and included serum neuron-specific enolase (NSE), brain-derived neurotrophic factor (BDNF), and homocysteine (Hcy). We performed enzyme-linked immunosorbent assays in the laboratory of Xiyuan Hospital to determine ET-1 (KE1362, 96T, ImmunoWay Biotechnology, Plano, TX, USA), VEGF (KE00085, 96T, ProteinTech, Richmond, CA, USA), vWF (ELH-VWF-1, 1*∗*96-VELL, RayBiotech, Peachtree Corners, GA, USA), and BDNF (KE00096, 96T, ProteinTech) serum levels. We used a chemical detection method to assess serum NO (ab65328, 2*∗*96T, Abcam, Cambridge, UK). Serum NSE levels were determined by the electrochemiluminescence method, using the Roche Cobas e601 device (Roche Diagnostics GmbH, Mannheim, Germany) and commercial kits (Cat.12133113122, Roche Diagnostics GmbH). Hcy levels were determined by an enzymatic method using the Roche Cobas e701 device (Roche Diagnostics GmbH, Mannheim, Germany). All the aforementioned protocols were conducted according to the manufacturer's instructions.

### 2.6. Outcomes

The primary outcomes were the neuropsychology scale scores and serum indexes associated with neurological and vascular endothelial function. Specially trained researchers assessed the patients' pre- and posttreatment MMSE, NIHSS, and Chinese Medicine Symptom Scale (CM-SS) scores. The evaluation criteria for the clinical efficacy rate for the MMSE and NIHSS were as follows: efficacy index = (pretreatment integral − posttreatment integral)/pretreatment score × 100%. An efficacy index ≥85% indicated a control case; from ≥50% to <85% indicated an obvious effect case; from ≥20% to <50% indicated an effectiveness case; and <20% indicated an ineffectiveness case. Deterioration was indicated by an efficacy index > −20%. Efficacy rate = control + obvious effect + effectiveness [29].

Secondary outcomes were the activities of daily living (ADL) scale scores and the safety indices in the Community Health Service Center of Yanqing, Beijing. Safety outcomes were nausea/vomiting and allergic reactions that disappeared soon after stopping the supplements. Furthermore, we recorded any adverse events during the treatment course.

### 2.7. Follow-Up

We examined the participants as outpatients every one-and-a-half months. The course of the intervention was 12 weeks. Twenty-four patients dropped out during the study, while 172 patients completed the trial, of which 85 patients were in the SMYZF group and 87 patients in the Ginkgo group.

### 2.8. Statistical Analysis

We included 172 participants who completed the trial in the final analysis. Lilliefors normality test was used to assess whether variables were normally distributed. Normally distributed data were analyzed using the two independent-samples' Student's *t*-test to determine between-group differences and descriptive statistics which were calculated for all variables (mean and SD). Nonnormally distributed data were analyzed using a nonparametric test (Wilcoxon test), and descriptive statistics were calculated as median and interquartile range. We analyzed continuous variables using the chi-square test (*χ*^2^ test) and categorical variables using the ridit test. Statistical significance was set at *P* value < 0.05. All statistical analyses were performed using SPSS 22 (IBM Corp, GSA ADP, US).

## 3. Results

### 3.1. Study Population

**Figure 1:**
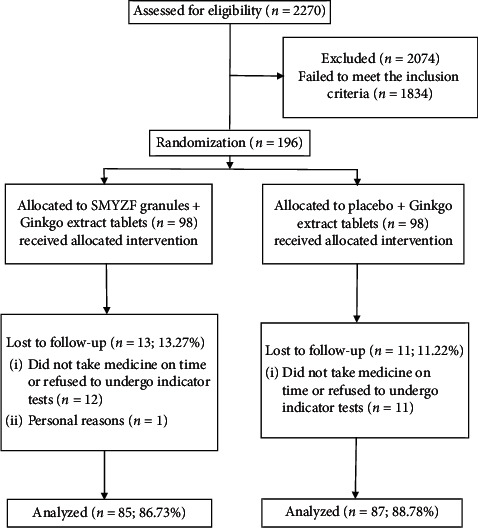
Trial profile.

Of the 172 patients with mild-to-moderate VaD from two Community Healthcare Centers, Yanqing District, Beijing, who completed the study from June 2018 to January 2019 (Figure 1), 85 were males and 87 were females, with ages 50–83 years (mean: 67.65 ± 6.96 years). The Ginkgo group comprised 87 participants (males, 46; females, 41) with a mean age of 68.07 ± 6.83 years; in this group, 13 patients had coronary heart disease, 23 patients had diabetes, and 72 patients had hypertension. The SMYZF group comprised 85 participants (39 males; 46 females) with a mean age of 67.22 ± 7.12 years; in this group, 9 patients had coronary heart disease, 27 patients had diabetes, and 71 patients had hypertension. There were no significant between-group differences in the baseline data (*P* > 0.05, Table 1).

### 3.2. Comparison of Posttreatment Curative Effects of the MMSE and NIHSS

The posttreatment curative effects of improvement in the MMSE scores in the SMYZF and Ginkgo groups were 49.4% and 34.5%, respectively. Furthermore, the curative effects of a decrease in the NIHSS score in the SMYZF and Ginkgo groups were 89.41% and 64.37%, respectively. The posttreatment curative effects were significantly higher in the SMYZF group (*P* < 0.05), as shown in Table 2.

### 3.3. Comparison of Cognitive Function and Cognitive Domain in the MMSE

There was a significant posttreatment increase in the MMSE scores in both groups (*P* < 0.05). There was also a significant posttreatment enhancement in time orientation, immediate memory, delayed recall, retelling, comprehension, attention, and calculation ability in both groups (*P* < 0.05). The delayed recall, retelling, and comprehension scores increased more significantly in the SMYZF group compared with the Ginkgo group (*P* < 0.05) (Table 3).

### 3.4. Between-Group Comparisons of the Pre- and Post-Vascular Endothelial Function

There was a significant posttreatment decrease in the serum ET-1 level and an increase in the serum VEGF level in both groups after treatment (*P* < 0.05). In addition, there was a significant posttreatment increase in the serum NO level and decrease in the serum vWF level in the SMYZF group (*P* < 0.05). However, the serum ET-1 level decreased, and serum NO level increased more significantly in the SMYZF group compared with the Ginkgo group after treatment (both *P* < 0.05). There was no between-group difference in the posttreatment improvement in the serum VEGF and vWF levels (all *P* > 0.05) (Table 4).

### 3.5. Comparison of Pre- and Posttreatment in the NIHSS and ADL Scores

**Figure 2:**
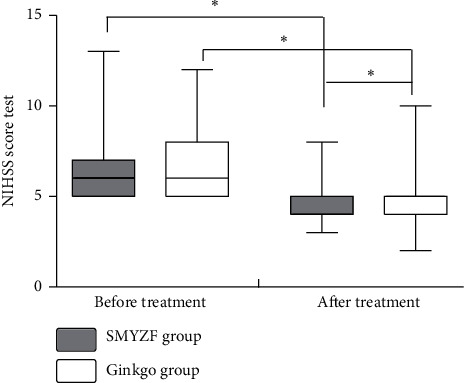
Comparison of NIHSS scores in the 2 groups. Note: ^*∗*^significant effect (*P* < 0.05).

**Figure 3:**
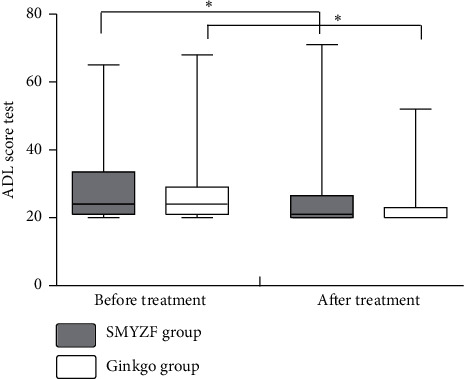
Comparison of ADL scores in the 2 groups. Note: ^*∗*^significant effect (*P* < 0.05).

There was a significant posttreatment decrease in the NIHSS and ADL scores in both groups (*P* < 0.05, Figures 2 and 3). Moreover, the posttreatment decrease in the NIHSS scores was greater in the SMYZF group compared with the Ginkgo group (*P* < 0.05, Figure 2). There was no significant posttreatment change in the ADL scores in either group (*P* > 0.05, Figure 3).

### 3.6. Serum Indexes Related to Neurological Function

Both groups showed a significant posttreatment decrease in the serum NSE and Hcy levels and an increase in the serum BDNF levels (all *P* < 0.05). However, the SMYZF group had significantly lower posttreatment serum NSE levels and higher serum BDNF levels than the Ginkgo group (both *P* < 0.05). There was no significant posttreatment change in the serum Hcy level in either group (both *P* > 0.05) (Table 5).

### 3.7. Comparison of Pre- and Posttreatment CM-SS Scores

There was a significant posttreatment decrease in the total CM-SS scores in both groups (both *P* < 0.05); moreover, the posttreatment total CM-SS scores were lower in the SMYZF group compared with the Ginkgo group. There was a significant posttreatment improvement in single symptoms, including a decline in intelligence, soreness, and weakness of the waist and knees, rigid and silent behavior, cardiopalmus, and tinnitus in both groups (all *P* < 0.05). Moreover, there was a significant posttreatment improvement in symptoms including restlessness, irritability, cyanosis of the lips, and nocturia in the SMYZF group (all *P* < 0.05); there was also a posttreatment improvement in the symptoms of spasm and numbness of limbs in the Ginkgo group (both *P* < 0.05). The SMYZF group showed a significant posttreatment improvement in the symptoms of decline in intelligence, restlessness, irritability, and cyanosis of the lips, as compared with the Ginkgo group (*P* < 0.05) (Table 6).

## 4. Discussion

In this study, we investigated the efficacy and safety of using SMYZF granules and Ginkgo extract tablets for the treatment of VaD. We found that SMYZF combined with Ginkgo extract tablets had curative effects on VaD, with improvement of cognitive, vascular endothelial, and neurological function; our findings also demonstrated the safety of this treatment.

VaD is caused by cerebral ischemia and is associated with various vascular risk factors including artery disease, cardiac thrombus embolism, hemodynamic changes, hemorrhage, hematological factors, and diabetes. Subsequently, it causes degeneration and necrosis of neurons and cognitive impairment [30]. Previous studies have reported that VaD treatment is mainly associated with cerebrovascular circulation improvement, increased blood flow, activated brain tissue metabolism, and promotion of neuronal cell functional recovery, thus mitigating dementia symptoms [31]. Previous experimental studies of SMYZF have reported that this herbal formula could reduce neuron damage in the hippocampus [19]. Furthermore, previous clinical studies have reported that SMYZF could improve vascular endothelium function, blood mobility, cerebral blood flow, cognitive function, and memory [20]. These findings, together with our own, demonstrate the efficacy of SMYZF in improving cognitive function in patients with VaD. In TCM, SMYZF, which comprises Radix et Rhizoma Ginseng, Rhizoma Gastrodiae, Ramuli Euonymi, and Rhizoma Chuanxiong, has been reported to replenish Qi, remove blood stasis, and calm the latent yang of the liver. Ginsenosides Rg1, Rb1, gastrodin, quercetin, and ferulic acid are the main bioactive components in SMYZF [32].

Research has demonstrated that ginsenosides, which are the main components of Ginseng, have protective effects on the vascular endothelium [33]. Ginsenosides have been reported to promote nerve cell regeneration, halt the deterioration of dementia, and improve the capacity for learning and memory [19]. To some extent, ginsenoside Rb1 could improve vascular endothelial function by improving inflammation, reducing oxidative stress, and increasing NO synthesis. Moreover, ginsenosides Rb1 and Rg1 have been reported to protect vascular endothelial cells from oxygen-glucose deprivation/reoxygenation injury and to promote nerve cell regeneration. Furthermore, they have been reported to interfere with the pathological dementia process and improve learning and memory [34, 35]. Gastrodin has been shown to increase the activity of cerebral microvascular endothelial cells after ischemic injury [36]. It can maintain the vasoconstriction balance by affecting the secretion of vasoconstrictive ET-1 by vascular endothelial cells [37]. Gastrodin regulates vasomotor and neurotransmitter functions, improves the blood supply of the nervous system, and prevents cerebral ischemia, inflammation, and amyloid protein injury, thus protecting nerve cells [36, 38]. Increasing NO levels and decreasing ET levels could exert a protective effect on vascular endothelial function [15, 39]. Previous experimental findings in murine studies have indicated that Ramuli Euonymi could improve hypoxia tolerance, relieve vasospasm, and repair damaged vascular endothelium [40]. Ligustrazine induces microvessel dilation and increases blood flow, which is beneficial to the secretion of vasoactive substances by vascular endothelial cells. Rhizoma Chuanxiong promotes the proliferation of microvascular endothelial cells and microvessels and promotes angiogenesis [41]. Previous experimental studies have reported that Rhizoma Chuanxiong could affect the apoptosis signal transduction system downstream of caspase and inhibit apoptosis of vascular endothelial cells by regulating Bcl-2 and caspase-3 gene expression [42]. Ferulic acid inhibits platelet aggregation and thromboxane release and has an antithrombotic effect. Ferulic acid also scavenges free radicals, regulates nerve cell homeostasis, and protects nerve cells. Hence, these characteristics of the active ingredients of SMYZF may underlie the positive effects noted in patients with VaD.

The MMSE is used worldwide to assess cognitive function [43], with higher MMSE scores indicating better cognitive function. We used the MMSE to assess orientation ability (time orientation, spatial orientation, and calculation), memory (immediate memory and delayed memory), linguistic ability (naming, comprehension, and retelling), comprehension, attention, and calculation ability. We found a posttreatment improvement in the aforementioned domains in both groups. Furthermore, the SMYZF group showed a significant improvement in retelling, linguistic comprehension, and delayed memory abilities compared with the Ginkgo group. These findings indicate that SMYZF could improve cognitive function in patients with VaD.

ET and NO play a crucial role in vasomotor functions. Currently, ET-1 is the most potent vasoconstrictor known, while NO is the most potent endogenous vasodilator [36, 39]. Many studies have reported a positive association between ET-1 levels and vascular cognitive impairment severity [44, 45]. ET-1-induced vasoconstriction causes lumen stenosis, cerebral ischemia, and hypoxia, which promote the development of dementia, which is closely associated with cognitive impairment [39]. Previous studies have reported that patients with VaD have significantly lower serum NO levels and impaired vascular endothelial function compared to healthy individuals, which negatively affects VaD prognosis. In this study, we found a significant posttreatment increase in the serum NO levels and decrease in the serum ET-1 levels; and these posttreatment changes were more significant in the SMYZF group.

These findings indicate that SMYZF combined with Ginkgo extract tablets could significantly improve cognitive function and reduce cognitive decline symptoms in patients with VaD. Moreover, the underlying mechanism might be related to the improvement of vascular endothelial function and the restoration of normal vasomotor state. We found a significant posttreatment increase in serum VEGF levels in both groups and a significant decrease in the serum vWF levels in the SMYZF group. VEGF can directly act on vascular endothelial cytokines, effectively enhancing small blood vessel permeability and promoting the metastasis of endothelial cells and stromal cells. Furthermore, high VEGF expression can alleviate neuronal injury caused by ischemia and hypoxia [46, 47]. vWF is a marker for endothelial cell injury and dysfunction [48]. More severe endothelial cell injury is associated with lower vWF levels in endothelial cell cytoplasm and higher serum vWF levels. Our finding of posttreatment increases in serum VEGF levels indicates the promotion of cerebral microvascular regeneration or inhibition of NO-mediated vascular endothelial cell secretion of ET and vWF, as well as improvement of the hypoxic state of brain tissue and cognitive function in patients with VaD. Our study findings thus indicate that SMYZF combined with Ginkgo extract tablets could attenuate vascular endothelial cell dysfunction in patients with VaD.

NSE and BDNF can regulate the growth, development, and regeneration of the nervous system. NSE is highly specific for nerves, and previous studies have reported a significant negative correlation between serum NSE levels and poststroke MMSE scores. NSE might be involved in the regulation of neuronal regeneration [49]. BDNF might improve learning and memory in the pathological state [50], which could play an important role in VaD pathogenesis and development. We found a significant posttreatment decrease in serum NSE levels and a significant increase in serum BDNF levels in both groups, with a greater improvement in the SMYZF group. The NIHSS is used to evaluate neurological function. There were significantly lower posttreatment NIHSS scores in the SMYZF group compared with the Ginkgo group in our study. Patients with VaD often present with decreased ADL. We found a significant posttreatment decrease in the ADL scores in both groups. These findings indicate that SMYZF combined with Ginkgo extract tablets improved neurological function of patients with VaD more than the Ginkgo extract tablets alone, and this effect may be related to vascular endothelium function improvement.

The etiology and pathogenesis of VaD is complex and is closely related to various cardiovascular and cerebrovascular diseases. In the early and middle stages of VaD, there is accompanying cognitive decline, such as memory and intelligence decline, as well as personality changes, such as restlessness, irritability, and neurological impairment. We found that the SMYZF group had lower posttreatment total CM-SS scores compared with the Ginkgo group. Furthermore, the SMYZF group exhibited significant improvement in the symptoms of mental retardation, restlessness, irritability, and lip cyanosis compared with the Ginkgo group. This demonstrates a significant clinical effect of SMYZF combined with Ginkgo extract tablets for improving the TCM syndromes of patients with VaD; these effects may be related to the improvement of vascular endothelial and cognitive function, as well as neurological function and protection of neurons.

This study had several limitations. First, the study area was limited to Yanqing, Beijing, which limits the generalizability of the findings to other regions. Second, the study participants were middle-aged and elderly and with lower education and economic levels, which might have affected the NIHSS and ADL scale scores. Finally, the use of multiple evaluators may have resulted in subjective differences.

## 5. Conclusion

SMYZF combined with Ginkgo extract tablets could improve vascular endothelial function, which might help improve cognitive and neurological functions in patients with VaD. Furthermore, these treatments can repair damaged nerves and improve symptoms of TCM syndrome in patients with mild-to-moderate VaD.

## Figures and Tables

**Table 1:** Patients' baseline characteristics.

	SMYZF group (*n* = 85)	Ginkgo group (*n* = 87)	*P* value
Age, years, mean (SD)	67.22 (7.12)	68.07 (6.83)	0.428
Sex, *n* (%)			0.359
Male	39 (46)	46 (53)	
Female	46 (54)	41 (47)	
Education level, *n* (%)			0.842
Below primary	7 (8)	8 (9)	
Primary	36 (42)	42 (48)	
Middle school	41 (48)	36 (41)	
High school or above	1 (1)	1 (1)	
BMI, kg/m^2^, mean (SD)	25.00 (3.57)	24.63 (3.34)	0.484
Heart rates, median (IQR)	70 (64–76)	70 (65–76)	0.510
Risk factors, *n* (%)			
Hypertension	71 (84)	72 (83)	0.893
Diabetes	27 (32)	23 (26)	0.442
Hyperlipidemia	19 (22)	14 (16)	0.297
Coronary artery disease	9 (11)	13 (15)	0.393
Scale scores, median (IQR)			
HAMD	5 (3–6)	5 (3–6)	0.820
HIS	9 (8–9)	8 (7–10)	0.300
CDR	1 (1–1)	1 (1–1)	0.977
NIHSS	6 (5–7)	6 (5–8)	0.315
ADL	24 (21–33.5)	24 (21–29)	0.666

Note: sex, education level, hypertension, diabetes, hyperlipidemia, and coronary heart disease history were analyzed by chi-square test (*χ*^2^ test).

**Table 2:** Comparison of MMSE and NIHSS scores after treatment in the 2 groups (*n*%).

Scale	*n*	Group	Control cases (%)	Obvious effect cases (%)	Effectiveness cases (%)	Ineffectiveness cases (%)	Total effective cases (%)	*P* value
MMSE	85	SMYZF group	2	6	34	42	49.4	0.041^*∗*^
	87	Ginkgo group	0	4	26	56	34.5	
NIHSS	85	SMYZF group	0	1	75	9	89.41	0.040^*∗*^
	87	Ginkgo group	0	5	51	31	64.37	

^*∗*^Significant effect (*P* < 0.05).

**Table 3:** Comparison of the MMSE before and after treatment in the 2 groups.

	SMYZF group (*n* = 85), median (IQR)	Ginkgo group (*n* = 87), median (IQR)	*P* value
Total scores			
Before treatment	20 (17–21.5)	20 (17–22)	0.955
After treatment	24 (21–26)	23 (18–26)	0.048^*∗*^
Orientation ability			
Time orientation			
Before treatment	4 (3–5)	4 (3–5)	0.162
After treatment	4 (3–5)	4 (4–5)	0.547
Spatial orientation			
Before treatment	5 (5–5)	5 (5–5)	0.561
After treatment	5 (5–5)	5 (5–5)	0.849
Memory ability			
Immediate memory			
Before treatment	3 (2–3)	3 (2–3)	0.956
After treatment	3 (3–3)	3 (3–3)	0.680
Delayed recall			
Before treatment	0 (0–2)	0 (0–1)	0.127
After treatment	2 (0–2)	1 (0–2)	0.046^*∗*^
Linguistic ability			
Naming			
Before treatment	2 (2–2)	2 (2–2)	0.312
After treatment	2 (2–2)	2 (2–2)	>0.999
Retelling			
Before treatment	0 (0–1)	0 (0–0)	0.182
After treatment	1 (1–1)	1 (0–1)	0.001^*∗*^
Comprehension			
Before treatment	2 (1–3)	2 (1–3)	0.941
After treatment	3 (3–3)	3 (2–3)	<0.001^*∗*^
Attention and calculation			
Before treatment	2 (1–3)	2 (1–4)	0.302
After treatment	3 (1–4)	3 (1–4)	0.954

^*∗*^Significant effect (*P*<0.05).

**Table 4:** Comparison of ET-1, NO, VEGF, and vWF before and after treatment.

	SMYZF group (*n* = 85), median (IQR)	Ginkgo group (*n* = 87), median (IQR)	*P* value
ET-1 (pg/mL)			
Before treatment	48.73 (24.86–96.48)	56.20 (31.22–146.48)	0.271
After treatment	22.04 (8.79–61.19)	30.85 (17.63–67.73)	0.043^*∗*^
NO (*μ*mol/L)			
Before treatment	16.06 (9.72–25.40)	15.14 (8.94–24.01)	0.614
After treatment	20.30 (11.26–36.82)	15.65 (9.04–26.86)	0.040^*∗*^
VEGF (pg/mL)			
Before treatment	128.37 (76.14–216.21)	130.99 (56.13–189.34)	0.207
After treatment	301.47 (187.20–447.45)	308.16 (196.57–477.22)	0.491
vWF (ng/mL)			
Before treatment	11643.45 (6466.81–15808.98)	9676.81 (6391.48–14450.25)	0.193
After treatment	9722.73 (6176.22–14078.82)	8913.02 (6278.56–13478.81)	0.385

^*∗*^Significant effect (*P*<0.05).

**Table 5:** Comparison of NSE, BDNF, and Hcy in the 2 groups before and after treatment.

	SMYZF group (*n* = 85), median (IQR)	Ginkgo group (*n* = 87), median (IQR)	*P* value
NSE			
Before treatment	7.42 (5.97–10.06)	7.75 (6.14–9.96)	0.877
After treatment	5.20 (4.23–6.08)	5.93 (4.65–7.55)	0.030^*∗*^
BDNF			
Before treatment	22.88 (14.79–32.70)	26.02 (17.52–32.86)	0.389
After treatment	38.79 (34.59–45.06)	35.78 (29.72–44.29)	0.026^*∗*^
Hcy			
Before treatment	8.87 (6.08–14.63)	9.62 (6.36–15.01)	0.595
After treatment	6.58 (4.83–9.95)	6.76 (3.89–12.5)	0.995

^*∗*^Significant effect (*P* < 0.05).

**Table 6:** Comparison of CM-SS scores in the 2 groups before and after treatment.

Symptom	SMYZF group (*n* = 85), median (IQR)	Ginkgo group (*n* = 87), median (IQR)	*P* value
Intelligence decline			
Before treatment	3 (3–6)	3 (3–6)	0.419
After treatment	0 (0–3)	3 (0–6)	0.006^*∗*^
Soreness and weakness of the waist and knees			
Before treatment	4 (2–4)	4 (2–4)	0.970
After treatment	2 (0–4)	2 (0–4)	0.570
Restlessness			
Before treatment	1 (0–2)	1 (0–2)	0.509
After treatment	0 (0–1)	1 (1–2)	<0.001
Rigid and silent			
Before treatment	0 (0–2)	0 (0–2)	0.792
After treatment	0 (0–2)	0 (0–0)	0.088
Spasm and numb of limbs			
Before treatment	1 (1–1)	1 (0–1)	0.531
After treatment	1 (0–1)	1 (0–1)	0.077
Cardiopalmus			
Before treatment	1 (0–2)	1 (0–1)	0.890
After treatment	0 (0–1)	0 (0–1)	0.909
Irritability			
Before treatment	1 (0–1)	1 (0–1)	0.380
After treatment	0 (0–1)	0 (0–2)	0.001^*∗*^
Dry skin			
Before treatment	0 (0–1)	0 (0–1)	0.615
After treatment	0 (0–1)	0 (0–1)	0.927
Cyanosis of the lips			
Before treatment	1 (1–2)	1 (1–2)	0.948
After treatment	1 (0–1)	1 (0–2)	0.013^*∗*^
Enuresis nocturna			
Before treatment	2 (1–2)	2 (1–2)	0.071
After treatment	2 (0.5–2)	2 (1–2)	0.713
Tinnitus			
Before treatment	1 (0–2)	1 (0–3)	0.166
After treatment	1 (0–1)	1 (0–2)	0.735
Total scores			
Before treatment	17 (11–20)	16 (11–21)	0.844
After treatment	10 (6–13)	11 (7–18)	0.033^*∗*^

^*∗*^Significant effect (*P* < 0.05).

## Data Availability

The dataset used and analyzed during the current study is available from the corresponding author (Hao Li) (e-mail: xyhplihao1965@126.com) on reasonable request.

## References

[1] Lopez A. D., Mathers C. D., Ezzati M., Jamison D. T., Murray C. J. (2006). Global and regional burden of disease and risk factors, 2001: systematic analysis of population health data. *The Lancet*.

[2] Dening T., Sandilyan M. B. (2015). Dementia: definitions and types. *Nursing Standard*.

[3] McGillc N. (2015). Experts: number of people with dementia worldwide expected to rise. *American Journal of Public Health*.

[4] Brookmeyer R., Kawas C. H., Abdallah N., Paganini-Hill A., Kim R. C., Corrada M. M. (2016). Impact of interventions to reduce Alzheimer’s disease pathology on the prevalence of dementia in the oldest-old. *Alzheimer’s & Dementia*.

[5] Anstey K. J., Peters R., Eramudugolla R., Jagger C., Peters R. (2019). A systematic review of meta-analyses that evaluate risk factors for dementia to evaluate the quantity, quality, and global representativeness of evidence. *Journal of Alzheimer’s Disease: JAD*.

[6] Fratiglioni L., Launer A. L., Jagger C. (2000). Prevalence of dementia and major subtypes in Europe: a collaborative study of population-based cohorts. Neurologic diseases in the elderly research group. *Neurology*.

[7] Rizzi L., Rosset I., Roriz-Cruz M. (2014). Global epidemiology of dementia: Alzheimer’s and vascular types. *BioMed Research International*.

[8] John T O. B. (2015). Vascular dementia. *Lancet (London, England)*.

[9] Wolters F. J., Ikram M. A. (2019). Epidemiology of vascular dementia. *Arteriosclerosis, Thrombosis, and Vascular Biology*.

[10] Jhoo J. H., Kim K. W., Huh Y. (2008). Prevalence of dementia and its subtypes in an elderly urban Korean population: results from the Korean Longitudinal Study on Health and Aging (KLoSHA). *Dementia and Geriatric Cognitive Disorders*.

[11] Chan K. Y., Wang W., Wu J. J. (2013). Epidemiology of Alzheimer’s disease and other forms of dementia in China, 1990–2010: a systematic review and analysis. *The Lancet*.

[12] Kalaria R. N., Maestre G. E., Arizaga R. (2008). Alzheimer’s disease and vascular dementia in developing countries: prevalence, management, and risk factors. *The Lancet Neurology*.

[13] Wu L., Walas S., Leung W. (2015). Neuregulin1-*β* decreases IL-1*β*-induced neutrophil adhesion to human brain microvascular endothelial cells. *Translational Stroke Research*.

[14] Ghanbarabadi M., Iranshahi M., Amoueian S., Mehri S., Motamedshariaty V. S., Mohajeri S. A. (2016). Neuroprotective and memory enhancing effects of auraptene in a rat model of vascular dementia: experimental study and histopathological evaluation. *Neuroscience Letters*.

[15] Castillo X., Castro-Obregón S., Gutiérrez-Becker B. (2019). Re-thinking the etiological framework of neurodegeneration. *Frontiers in Neuroscience*.

[16] Román G. C. (2002). Vascular dementia revisited: diagnosis, pathogenesis, treatment, and prevention. *Medical Clinics of North America*.

[17] Wollen K. A. (2010). Alzheimer’s disease: the pros and cons of pharmaceutical, nutritional, botanical, and stimulatory therapies, with a discussion of treatment strategies from the perspective of patients and practitioners. *Alternative Medicine Review:A Journal of Clinical Therapeutic*.

[18] Yakoot M., Salem A., Helmy S. (2013). Effect of Memo&reg;, a natural formula combination, on Mini-Mental State Examination scores in patients with mild cognitive impairment. *Clinical Interventions in Aging*.

[19] Li N.-N., Liu J.-G., Zheng R. (2019). Comparativestudy on pharmacological effects of Shenma Yizhi formula processed with three kinds of preparation techniques. *Beijing Journal of Traditional Chinese Medicine*.

[20] Wu Q., Liu F., Liu J. G., Wei Y., Liu M. X., Li H. (2017). Effects of shenma yizhi decoction on cognitive function and hemorheological state in mild and moderate vascular dementia. *Chinese Journal of Integrative Medicine on Cardio-Cerebrovascular Disease*.

[21] Porsalt R. D., Martin P., Lenègre A., Fromage S., Drieu K. (1990). Effects of an extract of Ginkgo Biloba (EGB 761) on “learned helplessness” and other models of stress in rodents. *Pharmacology Biochemistry and Behavior*.

[22] Serge G., Sandra S. (2014). Efficacy and tolerability of Ginkgo biloba extract EGb 761® in dementia: a systematic review and meta-analysis of randomized placebo-controlled trials. *Clinical Interventions in Aging*.

[23] Ni Y., Zhao B., Hou J., Xin W. (1996). Preventive effect of Ginkgo biloba extract on apoptosis in rat cerebellar neuronal cells induced by hydroxyl radicals. *Neuroscience Letters*.

[24] Hoyer S., Lannert H., Nöldner M., Chatterjee S. S. (1996). Damaged neuronal energy metabolism and behavior are improved by Ginkgo biloba extract (EGb 761). *Journal of Neural Transmission*.

[25] Kanowski S., Herrmann W. M., Stephan K., Wierich W., Hörr R. (1997). Proof of efficacy of the Ginkgo biloba special extract EGb 761 in outpatients suffering from mild to moderate primary degenerative dementia of the Alzheimer type or multi-infarct dementia. *Phytomedicine*.

[26] Mahboobi H., Golmirzaei J., Gan S., Jalalian M., Kamal M. (2014). Humanin: a possible linkage between Alzheimer’s disease and type 2 diabetes. *CNS & Neurological Disorders-Drug Targets*.

[27] Dubois B., Feldman H. H., Jacova C. (2007). Research criteria for the diagnosis of Alzheimer’s disease: revising the NINCDS-ADRDA criteria. *The Lancet Neurology*.

[28] Yang Y., Liu J.-p., Fang J.-y. (2019). Effect and safety of huannao yicong formula (还脑益聪方) in patients with mild-to-moderate Alzheimer’s disease: a randomized, double-blinded, donepezil-controlled trial. *Chinese Journal of Integrative Medicine*.

[29] Tian J.-Z., Han M. X., Tu J. W. (2000). Diagnosis, syndrome differentiation and therapeutic effect criteria of vascular dementia. *Journal of Beijing University of Traditional Chinese Medicine*.

[30] Nitkunan A., Barrick T. R., Charlton R. A., Clark C. A., Markus H. S. (2008). Multimodal MRI in cerebral small vessel disease: its relationship with cognition and sensitivity to change over time. *Stroke*.

[31] Iadecola C., Park L., Capone C. (2009). Threats to the mind: aging, amyloid, and hypertension. *Stroke*.

[32] Wu Q., Cao Y., Liu M. (2019). Traditional Chinese medicine Shenmayizhi decoction ameliorates memory and cognitive impairment induced by scopolamine via preventing hippocampal cholinergic dysfunction in rats. *Neuropsychiatric Disease and Treatment*.

[33] He F., Guo R., Wu S.-L., Sun M., Li M. (2007). Protective effects of ginsenoside Rb1 on human umbilical vein endothelial cells in vitro. *Journal of Cardiovascular Pharmacology*.

[34] Zhang J.-T. (2005). Nootropic mechanisms of ginsenoside Rg1--influence on neuronal plasticity and neurogenesis. *Acta Pharmaceutica Sinica*.

[35] Ni L., Liu B., Dluzen D.-E., Jin Y. (2007). Protective effects of ginsenoside Rg2 against glutamate-induced neurotoxicity in PC12 cells. *Journal of Ethnopharmacology*.

[36] Dai J.-N., Zong Y., Zhong L.-M. (2011). Gastrodin inhibits expression of inducible NO synthase, cyclooxygenase-2 and proinflammatory cytokines in cultured LPS-stimulated microglia via MAPK pathways. *PLoS One*.

[37] Hu J.-J., Hong Q.-T., Tang Y. P. (2001). Protective effects of gastrodine against lesions in cultured astrocytes caused by simulated cerebral ischemia and reperfusion, and its influence on the activity of nitric oxide synthase. *Journal of Beijing University of Traditional Chinese Medicine*.

[38] Walker D., Lue L. F., Beach T. G. (2001). Gene expression profiling of amyloid beta peptide-stimulated human post-mortem brain microglia. *Neurobiology of Aging*.

[39] Matsuzawa Y., Lerman A. (2014). Endothelial dysfunction and coronary artery disease. *Coronary Artery Disease*.

[40] Xiao D., Gu Z.-L., Bai J.-P., Wang Z. (1995). Effects of quercetin on aggregation and intracellular free calcium of platelets. *Zhongguo Yao Li Xue Bao*.

[41] Hou Y.-Z., Yang J., Zhao G.-R., Yuan Y. (2004). Ferulic acid inhibits vascular smooth muscle cell proliferation induced by angiotensin II. *European Journal of Pharmacology*.

[42] Chen R., Wu P., Cai Z. (2019). Puerariae Lobatae radix with chuanxiong rhizoma for treatment of cerebral ischemic stroke by remodeling gut microbiota to regulate the brain-gut barriers. *The Journal of Nutritional Biochemistry*.

[43] Manganelli F., Ragno M., Cacchiò G. (2008). Motor cortex cholinergic dysfunction in CADASIL: a transcranial magnetic demonstration. *Clinical Neurophysiology*.

[44] Briyal S., Philip T., Gulati A. (2011). Endothelin-A receptor antagonists prevent amyloid-*β*-induced increase in ETA receptor expression, oxidative stress, and cognitive impairment. *Journal of Alzheimer’s Disease*.

[45] O’Brien J. T., Erkinjuntti T., Reisberg B. (2003). Vascular cognitive impairment. *The Lancet Neurology*.

[46] Mahoney E. R., Dumitrescu L., Moore A. M., Cambronero F. E. (2019). Brain expression of the vascular endothelial growth factor gene family in cognitive aging and Alzheimer’s disease. *Molecular Psychiatry*.

[47] Spuch C., Antequera D., Portero A. (2010). The effect of encapsulated VEGF-secreting cells on brain amyloid load and behavioral impairment in a mouse model of Alzheimer’s disease. *Biomaterials*.

[48] Li Y., Zhang F., Nagai N. (2008). VEGF-B inhibits apoptosis via VEGFR-1–mediated suppression of the expression of BH3-only protein genes in mice and rats. *The Journal of Clinical Investigation*.

[49] Floerchinger B., Philipp A., Foltan M. (2014). Neuron-specific enolase serum levels predict severe neuronal injury after extracorporeal life support in resuscitation. *European Journal of Cardio-Thoracic Surgery*.

[50] Sakr H. F., Khalil K., Hussein A., Zaki M., Eid R., Alkhateeb M. (2014). Effect of dehydroepiandrosterone (DHEA) on memory and brain derived neurotrophic factor (BDNF) in a rat model of vascular dementia. *Journal of Physiology and Pharmacology*.

